# Current Integration of Tuberculosis (TB) and HIV Services in South Africa, 2011

**DOI:** 10.1371/journal.pone.0057791

**Published:** 2013-03-04

**Authors:** Joel C. Chehab, Amanda K. Vilakazi-Nhlapo, Peter Vranken, Annatjie Peters, Jeffrey D. Klausner

**Affiliations:** 1 Centers for Disease Control and Prevention, Pretoria, South Africa; 2 National Department of Health, Pretoria, South Africa; UNAIDS, Switzerland

## Abstract

**Setting:**

Public Health Facilities in South Africa.

**Objective:**

To assess the current integration of TB and HIV services in South Africa, 2011.

**Design:**

Cross-sectional study of 49 randomly selected health facilities in South Africa. Trained interviewers administered a standardized questionnaire to one staff member responsible for TB and HIV in each facility on aspects of TB/HIV policy, integration and recording and reporting. We calculated and compared descriptive statistics by province and facility type.

**Results:**

Of the 49 health facilities 35 (71%) provided isoniazid preventive therapy (IPT) and 35 (71%) offered antiretroviral therapy (ART). Among assessed sites in February 2011, 2,512 patients were newly diagnosed with HIV infection, of whom 1,913 (76%) were screened for TB symptoms, and 616 of 1,332 (46%) of those screened negative for TB were initiated on IPT. Of 1,072 patients newly registered with TB in February 2011, 144 (13%) were already on ART prior to Tb clinical diagnosis, and 451 (42%) were newly diagnosed with HIV infection. Of those, 84 (19%) were initiated on ART. Primary health clinics were less likely to offer ART compared to district hospitals or community health centers (p<0.001).

**Conclusion:**

As of February 2011, integration of TB and HIV services is taking place in public medical facilities in South Africa. Among these services, IPT in people living with HIV and ART in TB patients are the least available.

## Introduction

Tuberculosis (TB) remains the leading cause of death and morbidity among people living with HIV in developing countries [1], and HIV infection the most potent known risk factor associated with developing active TB [2]. According to UNAIDS, there are currently an estimated 33.3 million people living with HIV/AIDS and, in 2009, 1.2 million people were initiated on antiretroviral therapy for the first time. South Africa is home to the highest number of people living with HIV, has one of the highest TB incidence rates worldwide [3], and its TB/HIV co-infection rates are high, with as much as 80% of TB patients living with HIV in the province of KwaZulu-Natal [4]. Since 2009, the response to the HIV epidemic has accelerated in South Africa with a rapid scale up of HIV services, yet the expansion of TB services for people living with HIV has been slower, with only 26% of TB patients having been tested for HIV infection in 2009 [5], [6]. Isoniazid Preventive Therapy, has been shown to be effective in preventing active TB in people living with HIV [7], [8], [9], [10], [11], and is recommended by national guidelines in South Africa for all patients living with HIV where TB has been ruled out [12], yet remains poorly implemented at the country level [13]. In 2010, South Africa reported approximately 760,000 HIV-infected people having been screened for TB, and 120,000 of these having been started on IPT [14].

The profound impact that HIV and TB epidemics have had on health systems worldwide, particularly those in low-resource countries, has prompted the World Health Organization (WHO) to issue strategic guidelines for the integration of TB and HIV [15] in 2012 in order to combat both epidemics. Unlike the traditional approach where both diseases are tackled separately by the corresponding units in departments of health [16], these guidelines highlight WHO’s Three I’s- Intensified Case Finding (ICF), Isoniazid Preventive Therapy (IPT) and TB Infection Control (IC), as well as systematic testing and treatment of HIV in TB patients which all constitute key strategies to combat TB and HIV co-infection through an integrated delivery of these services [17].

South Africa’s Department of Health subsequently revised their TB management guidelines in 2009 [18] and Tuberculosis Prevention Therapy among people living with HIV in 2010, mandating that they be screened for TB, and recommending that those where active TB was ruled out be started on IPT [12]. South Africa adopted WHO’S policy on collaborative TB/HIV activities [15] including the Three I’s [17], early initiation of ART and speeding up the expansion of ART through decentralization and Nurse Initiation and Management of patients on ART (NIMART) [1], [19], [20], [21], [22]. No national survey had been done since the guidelines were revised to gauge the integration of TB and HIV services in South Africa.

We aimed to assess the current state of TB and HIV integrated services in South Africa.

## Study Population and Methods

### Study Population

We surveyed the TB and HIV focal persons of 49 public medical facilities in the high HIV prevalence districts in South Africa’s nine provinces from March 1^st^ to March 31^st^ 2011.

### Methods

We used a multistage sampling method. Firstly, we systematically selected the single district in each province with the highest HIV prevalence. We chose two districts in Kwazulu-Natal, as it had the highest HIV prevalence and double the number of districts of other provinces [23]. Secondly, we assigned a number to each sub-district within these high-prevalence districts and randomly selected one sub-district using the random number generator on stattrek.com. Thirdly, we assigned a number to all facilities per selected sub-district, and stratified them by facility type- community health center, primary health clinic or district hospital. We then used the random number generator to select a random sample of three primary health clinics, one community health center and one hospital per sub-district. This ratio was chosen to approximate the current distribution of public medical facilities in South Africa. The lists of facilities used for this sampling was provided by the South African National Department of Health.

The sample was chosen and we assessed both HIV and TB services. We determined the availability of the HIV services in each facility and stratified this by facility, facility type and province. HIV services we assessed included patient-initiated HIV counseling and testing (HCT), provider-initiated counseling and testing (PICT), WHO clinical staging of HIV, CD4 count, co-trimoxazole preventive therapy (CPT), and antiretroviral therapy (ART). We further investigated ART service delivery to determine whether the service was being provided on-site and who was initiating patients on the therapy: doctors only, nurses only, or both.

We determined the availability of the TB services and stratified them by facility, facility type and province. We investigated the following TB services: routine TB symptom screening, TB clinical diagnosis, TB treatment, and Isoniazid Preventive Therapy (IPT). Routine TB symptom screening was defined as clients being asked if they have any of the following symptoms: persistent cough for more than two weeks, fever for more than two weeks, night sweats, and unexplained weight loss (more than 5 kg in a month). If the answer to any of those questions was yes, the patient was defined as a person with suspected TB. The clinical diagnosis of these patients was followed by the collection and analysis of a minimum of two sputum samples for microbial laboratory confirmation of disease. We also determined whether the availability of IPT and TB services differed for pregnant HIV-infected women, and if TB services differed for patients with STIs.

Lastly, we collected routine TB and HIV monitoring data for the month of February 2011 from registers of sampled sites on newly registered TB patients that included the number of; patients newly diagnosed with HIV-infection; patients on whom CD4 T cell count was conducted; patients eligible AND initiated on ART; newly diagnosed HIV-infected patients screened for TB symptoms; newly diagnosed HIV-infected patients screened negative and positive for any TB symptom; newly diagnosed HIV-infected patients screened positive for TB symptoms and initiated on TB treatment; patients initiated on IPT among facilities providing IPT.

### Questionnaire

A standardized anonymous survey was created in English, which contained questions on various aspects of TB and HIV service integration, including service availability, location of service provision (whether or not offered on-site), and reporting and recording of routine data. The questionnaire was piloted in a hospital and primary health clinic prior to the assessment, with appropriate changes made.

### Data Collection

The Principal Investigator trained ten teams of interviewers in the use of the standardized questionnaire prior to the assessment. Each data collection team assessed 5 sites. Data collection took place from the 1^st^ to the 31^st^ of March 2011.

After obtaining informed consent, the two staff members each responsible for TB and for HIV services in each selected facility was interviewed and administered the questionnaire. All information reported by interviewees was systematically verified by examining and collecting routine TB and HIV data from facility registers.

### Data Analysis

Questionnaire data were entered into an Epi-Info 3.5.1 database (Centers for Disease Control and Prevention, Atlanta, GA) for analysis. Descriptive statistics were calculated and compared by province and facility type, and differences in proportions calculated using Fisher's exact test.

### Ethical Considerations

The Centers for Disease Control and Prevention determined that the collection of routine program data and its analysis for this study was a non-research activity in accordance to United States Federal regulations and waived IRB review. Further, the South African National Department of Health waived the need for local human subjects’ review.

## Results

### Facilities

We assessed integration of TB and HIV services in 49 public medical facilities in the highest HIV-burden districts in each of South Africa’s 9 provinces from March 1^st^ to March 31^st^ 2011.

### Routine Chart TB and HIV Data

Among the surveyed facilities, 2,512 patients were newly diagnosed with HIV in February 2011, and 1,913 (76%) of those were screened for TB symptoms. Of those screened for TB symptoms, 148 (8%) screened positive for at least one symptom, 1332 (70%) screened negative, and chart data were missing for 433 (23%). Of the 148 individuals who had any potential TB symptom, 125 (85%) were initiated on TB treatment and 23 (16%) were not. Of the 1,332 who screened negative for TB, 616 (46%) were initiated on IPT, whereas 716 (54%) were not.

In February 2011, 1,072 patients were newly registered TB patients among surveyed sites. Of those, 144 (13%) were already on ART prior to TB clinical diagnosis, and 451 (42%) were newly diagnosed as HIV infected. Of those 451 patients, 385 (85%) had CD4 T-cell counts done, 314 (70%) had been initiated on CPT, and 84 (19%) were initiated on ART.

### HIV Service Availability

Table 1 shows the availability of HIV services. Of the 49 participating facilities, 49 (100%) provided HIV counseling and testing, and CD4 T-cell counts. Provider-initiated counseling and testing and ART were the least available services, provided by 22 (45%) and 35 (71%) sites respectively. No associations were found between HIV services’ availability and facility type or province.

**Table 1:** Distribution of HIV services and TB services in selected sites by province (N = 49), South Africa, March 2011.

	HIV service n (%)						TB service n (%)			
Province	HIV Counseling & testing	Provider-initiated Counseling &Testing	CD4 count	WHO Clinical Staging	CPT	ART	Routine TB Symptomscreening	TB diagnosis	TB Treatment	IPT
**Eastern Cape**	5 (100%)	1 (20%)	5 (100%)	4 (80%)	5 (100%)	5 (100%)	5 (100%)	5 (100%)	5 (100%)	5 (100%)
**Free State**	5 (100%)	5 (100%)	5 (100%)	5 (100%)	5 (100%)	4 (80%)	5 (100%)	5 (100%)	5 (100%)	4 (80%)
**Gauteng**	5 (100%)	4 (80%)	5 (100%)	5 (100%)	5 (100%)	3 (60%)	5 (100%)	5 (100%)	5 (100%)	5 (100%)
**Kwazulu-Natal**	10 (100%)	1 (10%)	10 (100%)	7 (70%)	8 (80%)	7 (70%)	10 (100%)	9 (90%)	8 (80%)	9 (90%)
**Limpopo**	5 (100%)	3 (60%)	5 (100%)	5 (100%)	5 (100%)	3 (60%)	5 (100%)	5 (100%)	5 (100%)	1 (20%)
**Mpumalanga**	5 (100%)	1 (20%)	5 (100%)	5 (100%)	5 (100%)	3 (60%)	5 (100%)	5 (100%)	5 (100%)	3 (60%)
**North West**	5 (100%)	3 (60%)	5 (100%)	5 (100%)	5 (100%)	4 (80%)	5 (100%)	5 (100%)	5 (100%)	5 (100%)
**Northern Cape**	4 (100%)	4 (100%)	4 (100%)	4 (100%)	4 (100%)	2 (50%)	3 (75%)	3 (75%)	2 (50%)	3 (75%)
**Western Cape**	5 (100%)	0 (0%)	5 (100%)	5 (100%)	5 (100%)	4 (80%)	5 (100%)	5 (100%)	5 (100%)	0 (0%)

### ART Service Delivery

By facility type, antiretroviral therapy was offered in all (100%) district hospitals and community health centers, and absent in 14 (48%) of the 29 primary health clinics assessed. Compared to the other two facility types, primary health clinics were less likely to offer ART (p = 0.0002). Table 2 illustrates the distribution of methods of managing and initiating patients on ART among selected facilities offering ART (N = 35). Of the 35 facilities where ART was available, 32 (91%) reported the method of initiation and management of patients on ART. In 10 (31%) of those 32 facilities, only nurses initiated and managed patients on ART (NIMART), 10 (31%) reported that patients were initiated on ART solely by doctors and 13 (38%) sites reported that both doctors and nurses initiated and managed patients on ART. No association between nurse or doctor initiation and ART uptake was found.

**Table 2:** Method of Initiation and management of ART patients among facilities offering ART (N = 35), South Africa, March 2011.

Method	n (%)
**Nurse-initiated management of ART patients (NIMART)** **only**	10 (29%)
**Doctor-initiated and managed only**	10 (29%)
**Patients initiated and managed by both nurses and** **doctors**	12 (34%)
**Not specified**	3 (9%)

### HIV Service Recording and Reporting

Table 3 illustrates the recording and reporting of HIV services in our assessed sites. Of the 49 (100%) sites where CD4 T-cell counts were available, 46 (94%) recording that data in registers and of those, 40 (87%) reported the data to district and sub-district levels. No associations between province or facility type and recording and reporting of HIV services were found.

**Table 3:** Recording and reporting of HIV services in selected sites (N = 49) in South Africa, March 2011.

HIV service	(N = 49)		
	Offered	Recorded	Reported
HCT	100% (N = 49)	100% (N = 49)	96% (N = 47)
CD4 count	100% (N = 49)	94% (N = 46)	87% (N = 40)
CPT	96% (N = 47)	96% (N = 45)	89% (N = 40)
ART	71% (N = 35)	97% (N = 34)	94% (N = 32)

### TB Service Availability

Table 1 shows the availability of TB services among our selected facilities. Of 49 facilities, 30 (61%) offered all four TB services, and all (100%) offered at least one TB service. Little variation by province was observed in the availability of routine TB symptom screening, clinical diagnosis and treatment. IPT was offered by 35 (71%) of sites surveyed and was the least available TB service. IPT availability varied by province, omnipresent in all (100%) sites in North West, Eastern Cape and Gauteng, but only available in Mpumalanga (n = 2; 40%) and Limpopo (n = 1; 20%) and absent (0%) altogether in all 5 Western Cape sites. By facility type, IPT was available in 22 (73%) primary health facilities, 5 (56%) community health centers and 8 (80%) district hospitals.

### Services for Pregnant Women and STI Patients

Only 57% of 35 sites implementing IPT rendered the service to eligible pregnant women. When the client was specified as an STI patient, by facility type, 27 (90%) primary health clinics offered routine symptom screening, 26 (86.7%) TB diagnosis and 23 (76.7%) TB treatment; 9 (90%) of district hospitals offered routine TB symptom screening and TB diagnosis, and 8 (80%) offered TB treatment; and each of these services was offered by 8 (88.9%) community health centers. With the exception of IPT, provision of TB services among pregnant HIV-infected women and STI patients varied little by province. Table 4 shows the availability of TB services to pregnant HIV-infected and STI patients among our selected facilities. Neither province nor facility type associated with provision of any TB service.

**Table 4:** Availability of TB services to pregnant HIV-infected and STI patients in selected sites (N = 49) by province in South Africa, March 2011.

Province	TB service n (%)						
	Offered to pregnant HIV Women				Offered to STI patients		
	Routine symptom screening	TB clinical diagnosis	TB treatment	IPT	Routine symptom screening	TB clinicaldiagnosis	TB treatment
**Eastern Cape**	5 (100%)	5 (100%)	5 (100%)	5 (100%)	5 (100%)	5 (100%)	4 (80%)
**Free State**	4 (80%)	4 (80%)	4 (80%)	2 (40%)	4 (80%)	4 (80%)	4 (80%)
**Gauteng**	5 (100%)	5 (100%)	5 (100%)	4 (80%)	4 (80%)	4 (80%)	4 (80%)
**Kwazulu-Natal**	10 (100%)	9 (90%)	7 (70%)	7 (70%)	10 (100%)	9 (90%)	7 (70%)
**Limpopo**	5 (100%)	5 (100%)	5 (100%)	0 (0%)	5 (100%)	5 (100%)	5 (100%)
**Mpumalanga**	5 (100%)	5 (100%)	5 (100%)	2 (40%)	4 (80%)	4 (80%)	4 (80%)
**North West**	5 (100%)	5 (100%)	5 (100%)	5 (100%)	4 (80%)	4 (80%)	4 (80%)
**Northern Cape**	3 (75%)	4 (100%)	3 (75%)	3 (75%)	3 (75%)	3 (75%)	2 (50%)
**Western Cape**	5 (100%)	5 (100%)	5 (100%)	0 (0%)	5 (100%)	5 (100%)	5 (100%)

## Discussion

Our study assessed the availability of integrated TB and HIV clinical services in a sample of public medical facilities in South Africa in March 2011. Our data show that integrated HIV and TB services are being provided in the public medical facilities in South Africa. Among the 49 sites we sampled, about three quarters of newly diagnosed HIV-infected patients were screened for TB symptoms. According to South African national data reported to the WHO, 58% of newly diagnosed HIV-infected people were screened for TB symptoms in South Africa in 2010 [14]. Most newly diagnosed HIV-infected patients who screened positive for any TB symptom in February 2011 among our 49 selected facilities were started on TB treatment. No data on the proportion of those patients who had laboratory-confirmed TB was collected, and our findings on initiation of TB treatment may suggest over-diagnosis of active TB on the basis of clinical findings alone. Less than half of the newly registered TB patients in February 2011 were diagnosed as being HIV infected, compared to 60% of tested TB cases being reported as HIV-infected in South Africa in 2010 [14], and of those, most had CD4 T-cell counts done that month.

Several interventions and programs implemented recently might account for those improvements. Firstly, the South African National Department of Health launched several national campaigns, including the Kick TB campaign in partnership with Desmond Tutu TB center and the University of Stellenbosch in December 2009, aimed at increasing TB and HIV awareness among school-aged children, reaching almost 39,000 learners to date [24], and the HIV Counseling and Testing campaign in April 2010, which has tested 10.2 million South Africans for HIV infection [25]. In 2010, South Africa renewed its commitment to uphold WHO’s policy on collaborative TB/HIV activities including the three I’s, and increased the CD4 T-cell count threshold for initiation of TB patients on ART from 200 cells/mm^3^ to 350 cells/mm^3^ [26].Those campaigns greatly increased advocacy for integrated TB/HIV services and necessitated increased collaboration between national, provincial and district health authorities which together could have positively affected TB and HIV service delivery in surveyed facilities. Secondly, the National IPT guidelines were revised and disseminated in 2010 [12], and the availability of guidelines has been shown to increase IPT uptake [27].

Not all TB and HIV services, however, are improving. In our study, the proportion of newly diagnosed TB patients living with HIV initiated on co-trimoxazole in February 2011 was lower than national estimates in South Africa of 74% in 2010 [14]. Additionally, less than a quarter of eligible newly registered TB patients newly diagnosed with HIV in February 2011 were initiated on ART, well below the reported 30% of TB patients living with HIV on ART in 2009 [28]. Further, less than half of eligible patients of sampled sites were initiated on IPT in February 2011, below the target of 60% set by the South African government for 2011–2012 [28].

South Africa is shifting from a vertical programmatic approach with separate staff and service model in the early 2000s [29], [30] to a decentralized integrated approach with a strengthening of primary healthcare services. The primary health clinics are empowered through official policies to become the main mode of health care delivery, including essential TB and HIV services such as TB diagnosis and treatment, IPT and NIMART [31]. Our study does show signs that decentralization is taking place with NIMART being used as a method of initiating and managing patients on ART in more than half of the ART-providing facilities. Among the 49 public medical facilities we assessed, however, IPT and ART uptake remain low. In addition, we found that primary health clinics were less likely to offer ART compared to other facility types, suggesting that increased ART coverage as a result of decentralization remains an ongoing process. With regards to low IPT uptake, the persistence of known barriers to IPT implementation, including fear of the selection of INH-resistant TB [32], [33], could explain the slow IPT uptake among the selected facilities.

Recommendations on ART initiation among TB patients have been a moving target: until recently, South African ART guidelines followed the 2003 WHO guidelines recommending the delay of ART initiation among co-infected patients with CD4 T-cell counts of 200 cells/mm^3^ or above until completion of TB therapy [34]. The newest ART initiation guidelines in TB patients now recommend starting ART after 2 to a maximum of 8 weeks of TB treatment in all TB patients living with HIV with CD4 T-cell counts of 350/mm^3^ or less. Despite those recent and significant changes in policy, barriers in ART initiation in TB patients living with HIV remain including concerns of immune reconstitution syndrome and drug-drug interactions [35], and could in part account for the low proportion (19%) of eligible co-infected patients in surveyed sites initiated on ART. The delay in ART initiation is worrisome, particularly in view of the findings of a recent trial in South Africa, which found that mortality was reduced by 56% among patients started on ART during TB treatment as compared to those initiated after the end of TB treatment, with no significant risk of increased adverse events [36].

Another barrier to the implementation of integrated services is under-staffing [37], which remains an issue in South Africa for several reasons. Firstly, there is a national shortage of both nurses and doctors. Secondly, there is uneven distribution of current staffing resources. A recent study revealed that staffing shortages particularly affect rural areas and public medical facilities, and that one of the biggest staffing issues was the inequities in distribution of essential practitioners between provinces, rural and urban areas and between public and private sectors [38], [39]. Overall, our study shows that both TB and HIV services are being provided at all levels of the public health care system, including the clinic level. These findings illustrate the progress the South African government is making towards a decentralized health care system [31].

### Limitations

We conducted the rapid assessment only in districts with the highest antenatal HIV prevalence, and our results might therefore not be representative of areas with lower HIV prevalence. Our assessment only included a few facilities in each province, thus limiting the generalizability of our results. Our small sample size reduced the power of the survey, and may have concealed statistically significant differences in our study population. A larger sample size could have shown an association between factors such as facility type and location of ART provision, and facility type and method of initiation and management of ART. We sought to maximize external validity through random sampling and by selecting sites based on the approximated national distribution of facility types. We randomly selected double the number of sites in Kwazulu-Natal to account for the fact that the province had double the number of districts compared to most other provinces and the highest overall HIV prevalence in 2008 [23]. Northern Cape only had four study sites as two of the facilities initially selected merged into a single entity shortly before data collection. Further, all selected facilities were public medical facilities as we were assessing the implementation of a national public medical program, and our results might not be representative of TB/HIV integration in private sector health facilities. We were unable to collect data on the number of patients screened positive for TB symptoms for whom TB was laboratory-confirmed. Finally, we did not collect data on HIV testing and prior knowledge of HIV status or the number of patients eligible for IPT completion in February 2011 among surveyed sites.

### Conclusion

Our study demonstrated important progress is being made towards integration of TB and HIV services in South Africa, where nearly all facilities offered routine TB screening to people living with HIV infection, and routine HIV Counseling and Testing to TB patients. However, uptake of other essential services, such as ART and IPT, needs to be improved, as less than half of eligible people living with HIV were initiated on IPT, and only a small proportion of newly registered TB patients newly diagnosed as HIV-infected in February 2011 were initiated on ART. Addressing those gaps is a priority and future interventions should build on existing efforts to support current national policies of routine TB screening of all HIV patients, initiation of all eligible HIV-infected patients on IPT and early ART initiation of eligible TB patients irrespective of CD4 T-cell count [40]. Our findings represent estimates of point prevalence and repeated surveys should be conducted to monitor trends over time.
